# New Forehead, New Confidence: Flap Advancement Post-cancer Excision

**DOI:** 10.7759/cureus.81458

**Published:** 2025-03-30

**Authors:** Gowthaam Ramesh, Manimaran R, Jesu Pencilin Yesuvadiyan, Karthikeyan Selvaraj, Srinivasan C

**Affiliations:** 1 General Surgery, Sree Balaji Medical College and Hospital, Chennai, IND; 2 Plastic Surgery, Sree Balaji Medical College and Hospital, Chennai, IND

**Keywords:** advancement flap, basal cell carcinoma (bcc), cosmetology, head and neck neoplasms, surgical case reports

## Abstract

Basal cell carcinoma (BCC) often affects sun-exposed regions, such as the forehead. Surgical excision with histopathologically confirmed clear margins remains the gold standard for treating BCC, especially for high-risk or recurrent cases. Wide local excision ensures complete tumor removal while minimizing the risk of recurrence. However, reconstructing defects in the forehead poses unique challenges due to limited excess skin, high tension, and the presence of muscles. Reconstruction strategies must prioritize preserving functional and cosmetic outcomes, including motor and sensory nerve integrity, and maintaining proper camouflaging incision lines and eyebrow position in relaxed skin tension lines (RSTLs). For large defects, a combination of multiple local tissue flaps is often favored over skin grafting, as it restores facial contours with adjacent, similar tissue and yields higher success rates with fewer complications. This report discusses a case of wide local excision of a BCC over the forehead, followed by defect reconstruction using advancement flaps from the temporal regions.

## Introduction

The term "rodent ulcer," now recognized as "basal cell carcinoma" (BCC), was primarily introduced by Jacob Arthur in Dublin in 1827 [1]. This name comes from the fact that the epithelial tumor cells resemble normal skin basal cells. The basal layer of the epidermis, along with its appendices, is the origin of this slowly spreading, locally invasive cancer. This condition is recognized as the most prevalent skin cancer among adults with fair complexions [2]. The face is highly prone to BCC due to regular exposure to sunlight. The forehead, cheeks, nose, and periocular region are most commonly affected [3]. Reconstructing the affected area after excision can be complex, especially when the cancer has invaded far into surrounding tissues, bone, or skin [4]. The incidence of BCC has increased considerably in the last 10 years and exhibits geographical variations [5]. Extended ultraviolet (UV) light exposure is the primary reason for BCC development [6]. Additional predisposing factors include scars, radiotherapy exposure, albinism, arsenic exposure, burns, immunosuppression, and genetic conditions such as Gorlin and Bazex syndromes [7-10]. The forehead, comprising one-third of the face, presents unique challenges for reconstruction due to its relatively high tension and limited excess skin available for use. Moreover, the forehead houses various critical structures and muscles. Therefore, preserving motor and sensory nerve functions is a key surgical objective. Essential considerations during forehead reconstruction surgeries include maintaining proper eyebrow positioning and concealing incision lines in relaxed skin tension lines (RSTLs), cosmetic subunit junctions, and hair-bearing areas [11]. Primary closure is the simplest approach to defect reconstruction. However, alternative techniques, such as second-intention healing, skin grafting, or local tissue flaps, may be used due to a lack of feasibility. These methods are particularly important for managing large defects requiring a combination of techniques for optimal outcomes. Local tissue flaps are generally preferred over skin grafts as they maintain the integrity of facial cosmetic units by aligning scars along their junctions and repairing them with adjacent, analogous tissue. Flaps also tend to possess higher success rates and fewer complications than skin grafts. The disfigurement and functional limitations caused by scar contractures, including landmark defects and malposition, can significantly impact a person's well-being and quality of life [12]. This case discusses about the management of BCC for a 50-year-old woman by wide local excision for bilateral advancement flap.

## Case presentation

**Figure 1:**
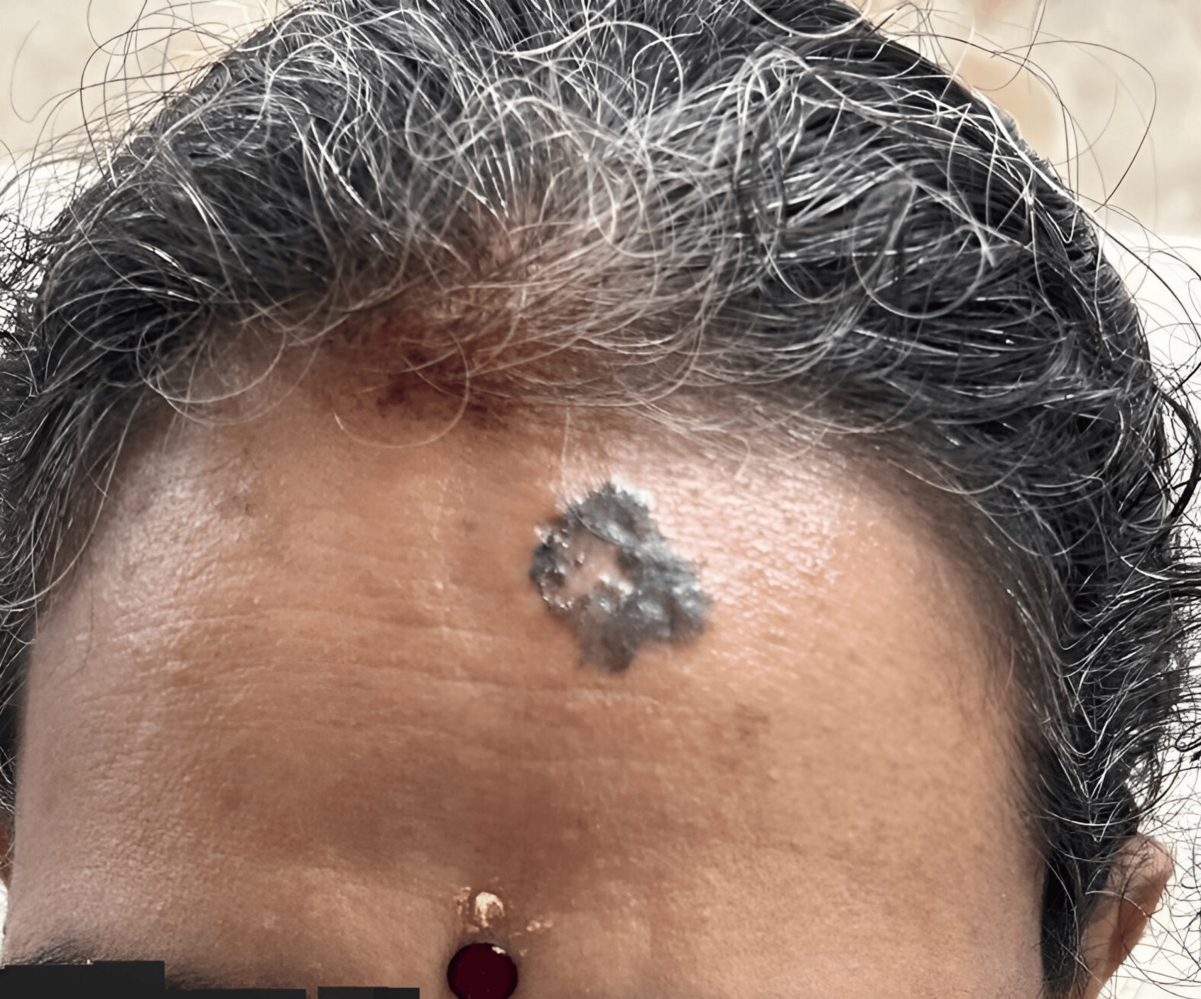
Preoperative image of basal cell carcinoma over the forehead

A 50-year-old Indian female patient came to the surgery outpatient department (OPD) with the complaint of a black pigmented lesion over her forehead for seven years. It was insidious in onset, slowly increased to attain the current size, and was not associated with pain or discharge. The patient had no other complaints and no known comorbidities. She revealed a history of excisional biopsy for a sebaceous cyst in the upper back two years ago. Physical examination revealed a 3 × 2 cm pigmented lesion on the forehead; the lesion was irregularly shaped with everted edges and did not bleed on touch. Induration was present, and the surrounding skin appeared normal. No palpable regional lymph nodes were noted (Figure 1).

**Figure 2:**
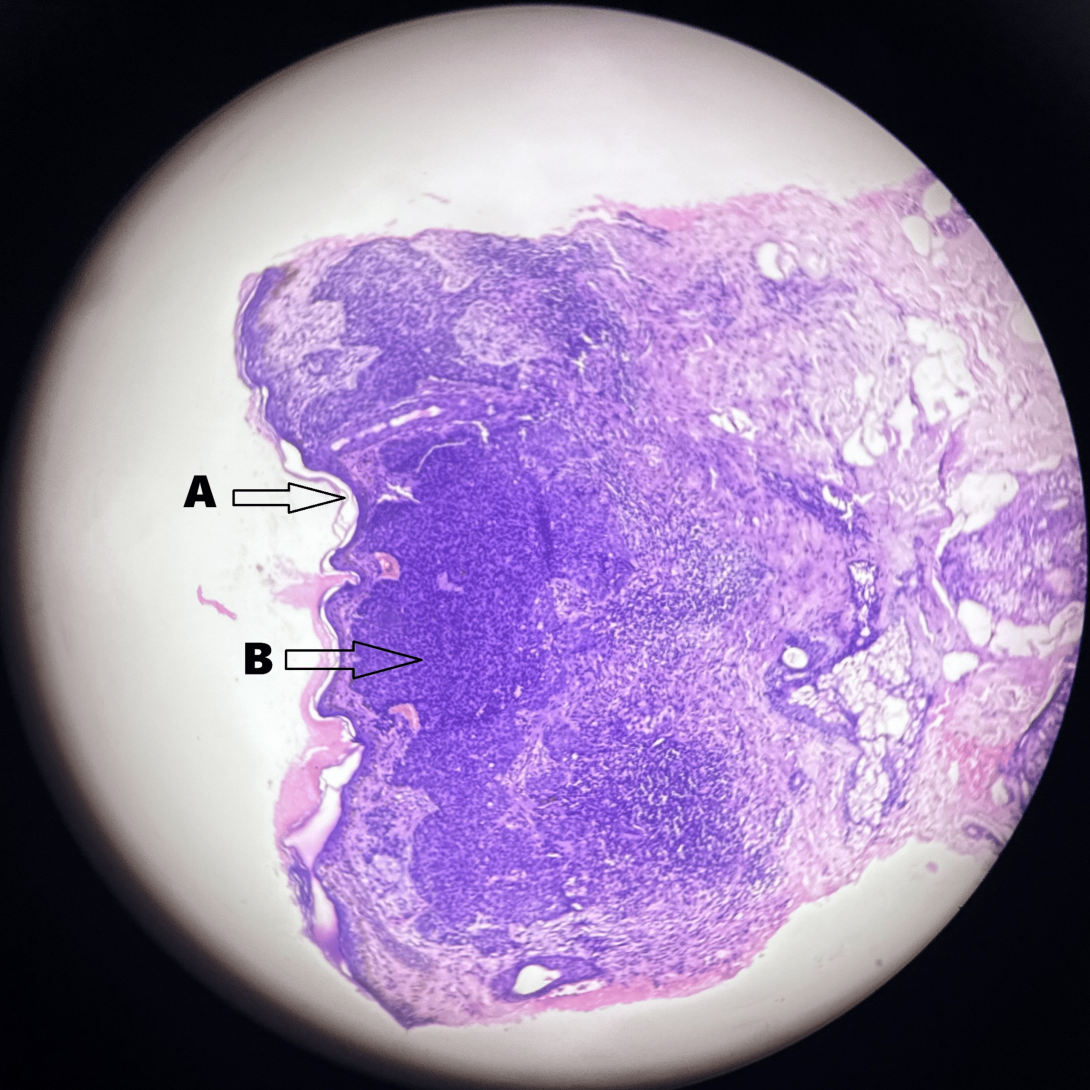
Edge wedge biopsy slide image of basal cell carcinoma Hematoxylin and eosin stains were used with the magnification being 10×. (A) Basaloid cells in the form of nests and cords. (B) Peripheral palisading

An edge wedge biopsy showed skin proliferation of basaloid cells in the form of nests and cords with peripheral palisading and no nuclear pleomorphism or increase in mitosis. Increased pigmented cells were also present (Figure 2).

**Figure 3:**
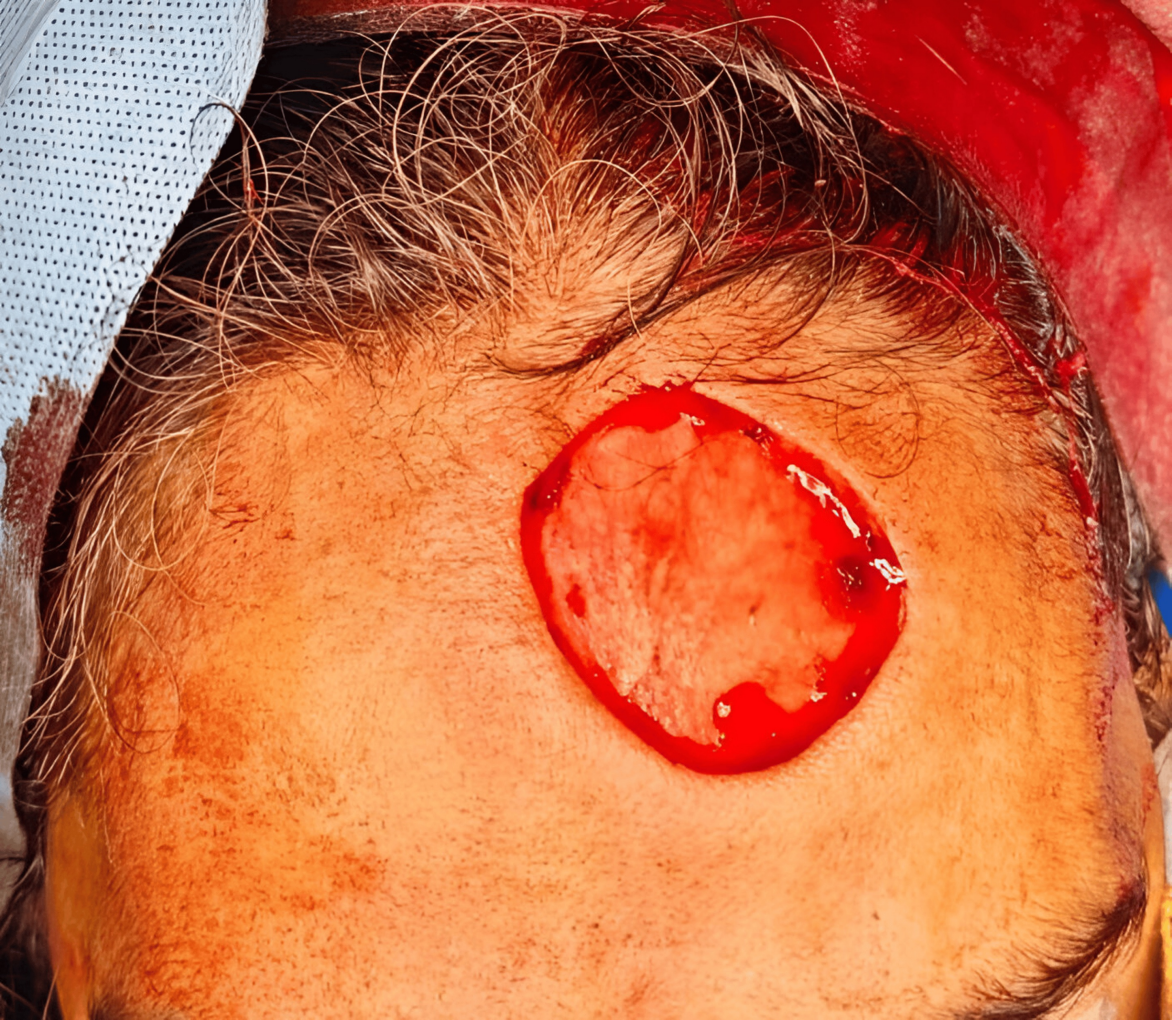
Wide local excision of basal cell carcinoma: intraoperative picture

**Figure 4:**
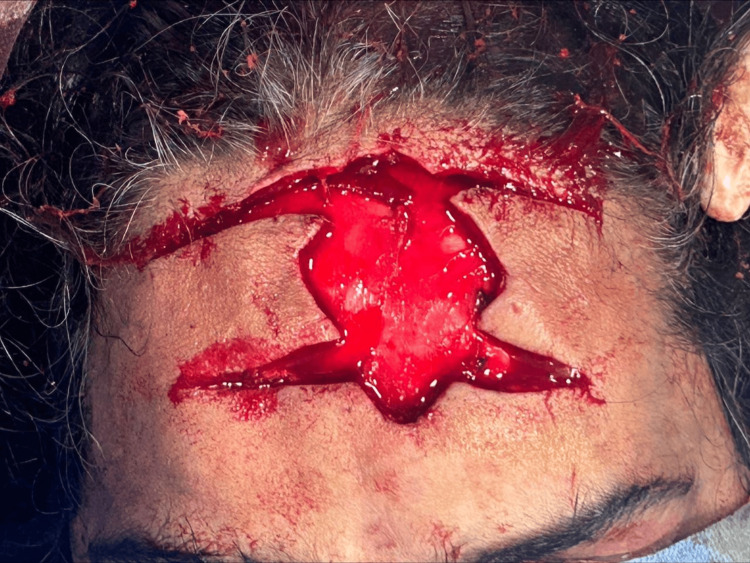
Wide local excision of basal cell carcinoma: intraoperative picture

The treatment plan included wide local excision of the BCC followed by reconstruction. A 0.5 cm margin was taken from the maximum tumor induration site, and the incision was deepened to the periosteal layer (Figure 3 and Figure 4).

**Figure 5:**
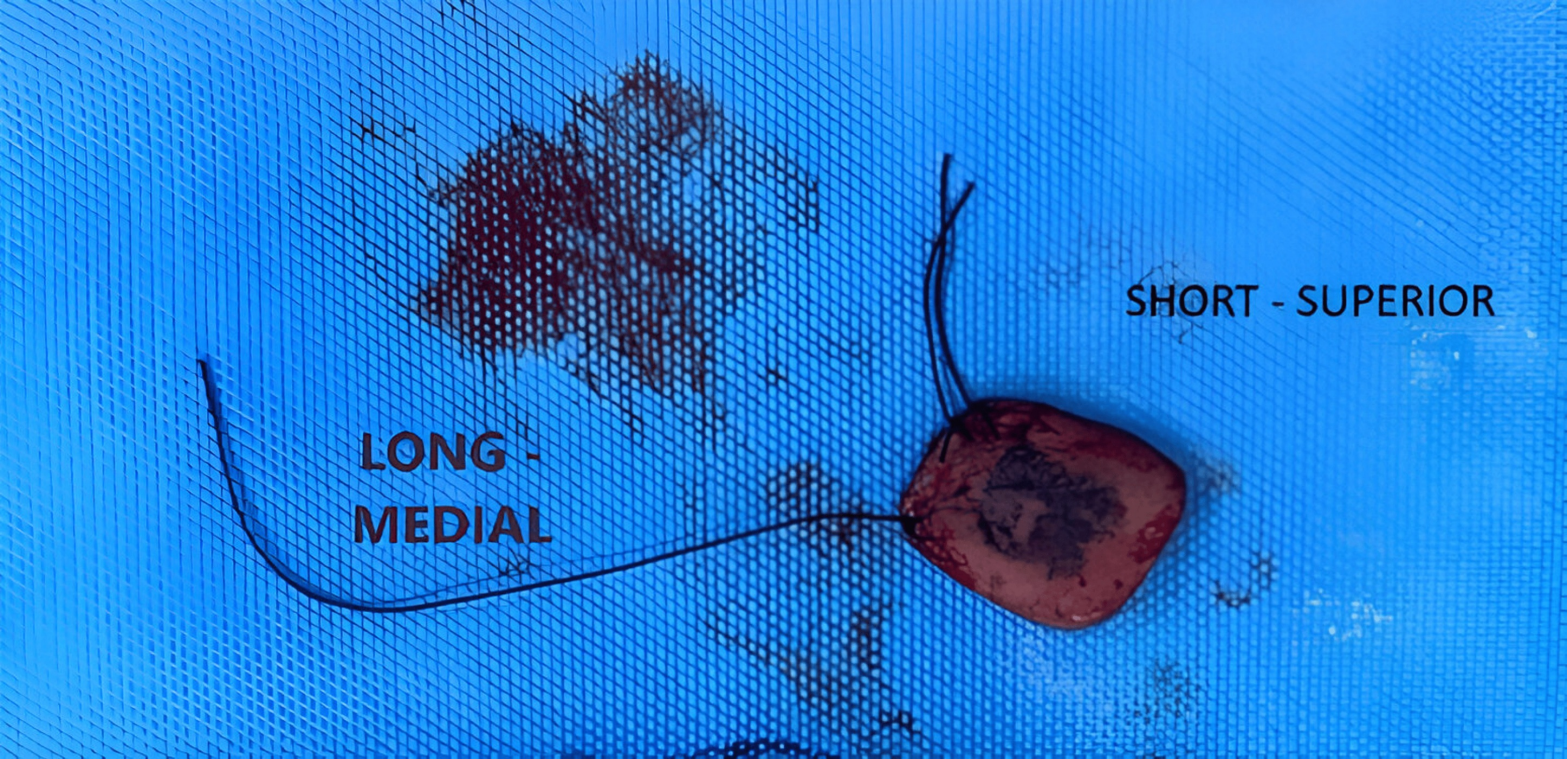
Specimen marking

The long thread was marked in the specimen as the medial margin, and the two short threads were marked as the superior margin (Figure 5).

**Figure 6:**
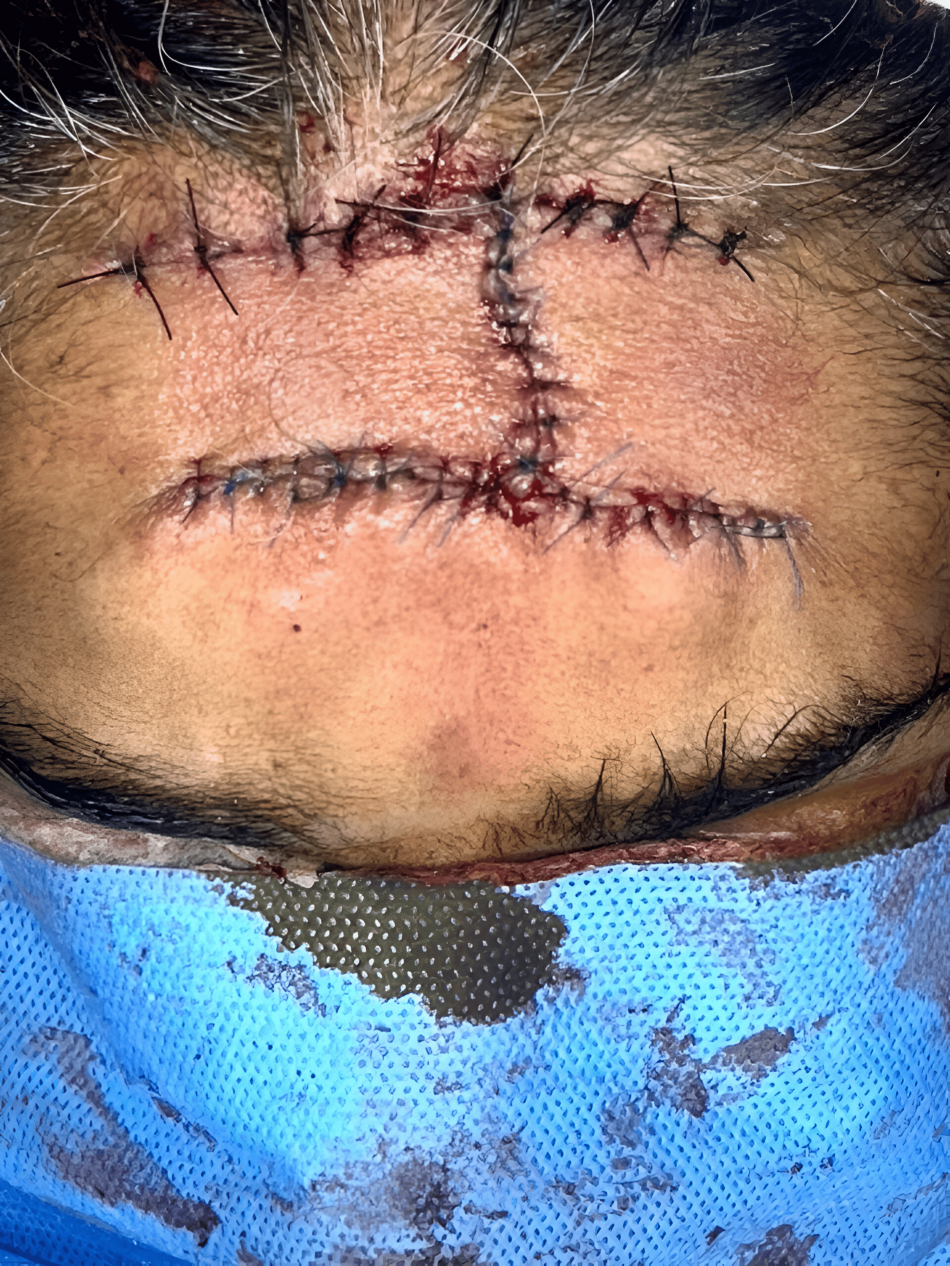
Bilateral advancement flap: intraoperative picture

The frozen section showed that all resected tumor margins were tumor-free. A bilateral advancement flap was done (Figure 6). 

**Figure 7:**
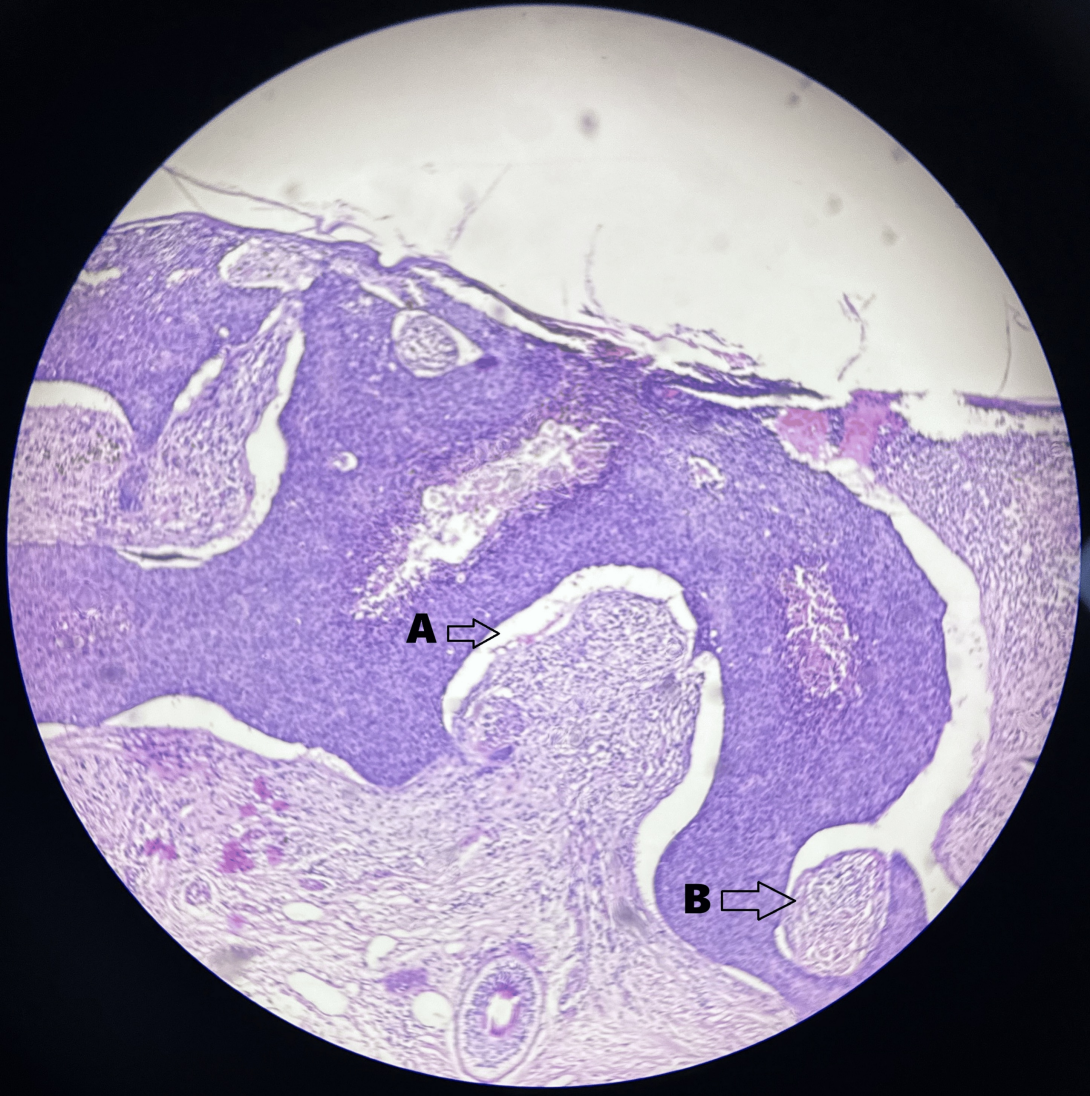
Histopathological image of basal cell carcinoma Hematoxylin and eosin stains were used with the magnification being 10×. (A) Basaloid cells in the form of nests and cords. (B) Peripheral palisading

In the histopathologic examination, the skin showed grey-black proliferating basaloid cells arranged in nests and cords, displaying peripheral palisading with no evidence of nuclear pleomorphism or increased mitotic activity. Pigmented BCC of the forehead was confirmed (Figure 7).

**Figure 8:**
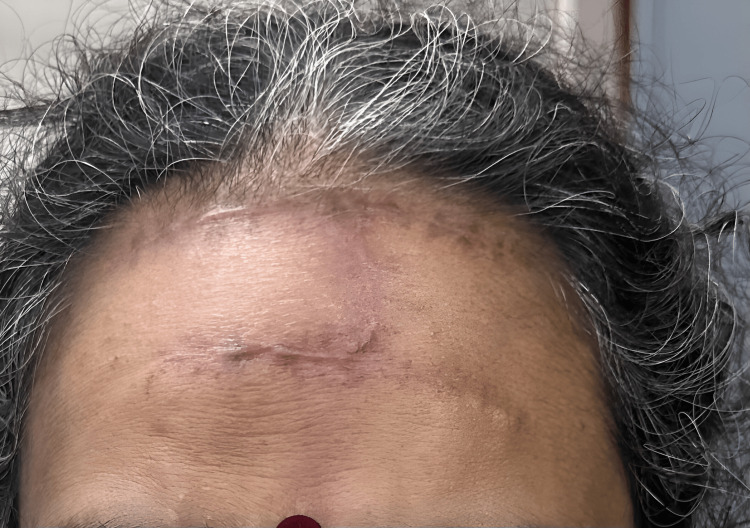
Follow-up picture

The patient was followed up for three months and had no complications (Figure 8).

## Discussion

BCC is the most frequently found skin cancer, with increasing incidence worldwide due to heightened UV light exposure and ozone layer depletion caused by environmental and industrial pollution. UV light exposure induces DNA damage, contributing to the formation of skin cancer. Moreover, individuals with immunosuppression are at a higher risk of developing nonmelanoma skin cancers. BCC is related to Rombo syndrome, Rasmussen syndrome, nevoid BCC syndrome, Bazex syndrome, and Darier's disease, often linked to mutations in the sonic hedgehog pathway. Several factors need consideration when selecting the appropriate treatment for BCC, including tumor size and stage, infiltration into adjacent tissues, location, general health of the patient, and history of prior treatments. Surgical removal remains the gold standard for treating BCC to eliminate the tumor while preserving both practical and cosmetic results. Surgical excision on the head generally achieves a 97% five-year lesion cure rate under 6 mm, along with 92% for lesions over 6 mm. Reconstructing large defects may necessitate multi-staged processes involving complex and multiple flap designs aligned with RSTLs. In this case, a forehead defect was addressed using bilateral advancement flaps, utilizing the frontal region as a donor site to maintain the integrity of facial cosmetic units by reestablishing contours with nearby, comparable tissue and positioning scars at their intersections. Research by Wang et al. supports the use of advancement flaps for managing large defects, as they offer higher success rates by reducing the risk of wound implications, tissue necrosis, and excessive tension [11,12].

## Conclusions

BCC is a frequent yet disfiguring kind of skin cancer that might require significant repair and excision if not addressed promptly. Early detection and treatment are essential in reducing the need for aggressive interventions. Educating patients on sun exposure prevention and routine skin examinations is critical for prompt identification and BCC management. Treatment plans should be tailored to each patient, accounting for lesion size, location, and depth, in addition to accessible donor esthetic subunits, the patient's overall health, and personal preferences.
